# A new approach to expert reviewer detection and product rating derivation from online experiential product reviews

**DOI:** 10.1016/j.heliyon.2021.e07409

**Published:** 2021-06-29

**Authors:** Atiquer Rahman Sarkar, Shamim Ahmad

**Affiliations:** Department of Computer Science & Engineering, University of Rashahi, Rajshahi, 6205, Bangladesh

**Keywords:** Experiential product, Expert reviewer, Reviewer ranking, Product rating, Product ranking

## Abstract

Consumer reviews have emerged as one of the most influential factors in a person's purchase behavior. The existing open-source approaches for detecting expert reviewers and determining product ratings suffer from limitations and are susceptible to manipulation. In this work, we addressed these limitations by developing two algorithms and evaluated them on three datasets from amazon.com (the largest dataset contains nearly eight million reviews). In the first algorithm, we used a combination of the existing open-source approaches such as filtering by volume of contribution, helpfulness ratio, volume of helpfulness, and deviation from the estimated actual rating to detect the experts. The second algorithm is based on link analytic mutual iterative reinforcement of product ratings and reviewers' weights. In the second algorithm, both reviewers and products carry weights reflecting their relative importance. The reviewers influence the product rating according to their weight. Similarly, the reviewers' weights are impacted by their amount of deviation from the estimated actual product rating and the product's weight. Our evaluation using three datasets from amazon.com found the second algorithm superior to the other algorithms in detecting experts and deriving product ratings, significantly reducing the avg. error and avg. Mean Squared Error of the experts over the best of the other algorithms even after maintaining similar product coverage and quantity of reviews.

## 1 Introduction

Customers are uncertain about the true quality of the products they want to buy and this uncertainty is greater in the case of experiential products like movies, books, songs because repeated purchase of the same product is quite uncommon for such products. When a potential customer seeks information regarding a product, most websites provide separate review text and aggregated rating for that product [1]. People spend hours going through numerous reviews to make an informed decision and build confidence in the product they are planning to buy. Several studies have shown that people cite product reviews as a top influence in their purchase behavior. In their study, Zhao et al. [2] have found evidence of "stronger learning from product reviews than learning from own experience" and this is more so in the cases of experiential products. McGlohon et al. [3] mentioned in their paper that consumer recommendations are the most credible form of advertising among 78% of survey responders (as cited in Nielsen, 2007). They also mentioned a BIGresearch survey (2009) which indicated that 43.7% of consumer electronics purchases are affected by word-of-mouth. Chen et al. [4] mentioned a Wall Street Journal report (2006) that 71% of online U.S. adults use consumer reviews for their purchases and 42% trust such a source. Askalidis and Malthouse [5] found that purchase likelihood increases by as much as 190% for low priced items and 380% for high priced items when product reviews are displayed. They also found that in the high availability of product reviews, users usually focus only on the first few reviews. Hu et al. [6] found that most customers use numerical ratings in the early stage of searching and awareness. Moreover, they also found that-as the customer move to the final stage of purchase, the associated text sentiments play an increasingly important role.

Several studies have empirically shown that positive reviews are associated with higher sales, whereas negative reviews tend to hurt sales of experiential products like books and movies [7]. As a result, some people try to manipulate/post fake reviews to influence customers. Zhao et al. [2] have shown that fake reviews create consumer uncertainty. Connors et al. [8] observed that reviewers who claim self-described expertise in their reviews get more helpfulness-votes by potential customers, although their reviews contain the same content (except the expertise-claim) as that of a regular reviewer. The trustworthiness of reviews and the reviewer's reputation play a vital role in influencing the user's purchase behavior [9, 10, 11]. It helps the user to distinguish information for making informed decisions [12].

These facts pose the question: using a dataset of reviews, could we find the expert reviewers, highlight their reviews and derive a product-rating representing the true rating of the product to help a customer? Our goal in this work is to address this issue and give consumers the chance to make a more informed decision when purchasing a product.

### 1.1 Types of reviews/rating scale used online

Common examples of rating scales used in eCommerce sites are the Likert scales [13] and the 1–5 rating scale in which a person selects the number which is considered to reflect the perceived quality of a product. Usually, online rating scales only allow one rating per user per product. However, some websites allow users to rate products in relation to several qualities. Most online rating facilities provide no or few qualitative descriptions of the rating categories. World's largest eCommerce sites such as amazon, eBay, aliexpress, Flipkart provide 1–5 rating scales for reviews. For example, Amazon uses the following attributes in their system-

• **Movie Id:** asin (Amazon Standard ID Number) e.g. amazon.com/dp/B00006HAXW
• **ReviewerId:** ID of the reviewer, e.g. A1RSDE90N6RSZF
• **ReviewerProfileName**: Name of the reviewer
• **Helpfulness:** count of thumb-ups and thumb-down received (fraction of users who found the review helpful)
• **Score:** rating of the product
• **Time:** time of the review (UNIX time)
• **Summary:** review summary
• **Text:** review-text

There are two important points to be noted about using these rating/review systems in the eCommerce sites. First, it is assumed that the ‘distance’ between the rating points is equal, i.e., equidistant. The perceived ‘distance’ between ratings 1 and 2 is the same as the perceived ‘distance’ between ratings 3 and 4. Secondly, a good rating/reviewing system will present symmetry about a midpoint. In such symmetric scaling, equidistant quality of attributes will easily be inferred. While a Likert scale is indeed ordinal, if we can ensure that the scale being used is symmetric and equidistant, then it will behave like an interval-level measurement. We can gain additional valuable information from these ratings if these two properties are ensured and we can perform the kinds of analysis appropriate for interval-level measurements. More about treating start-ratings and Likert-type scale like an interval-level measurement is discussed in section 4.6.

### 1.2 Problem statement

A review usually consists of the following minimal attributes: reviewer_ID, product_ID, product_rating, description, helpfulness, time_of_review. We define the problem statement as follows.

Given a set of reviews with the following attributes:

• reviewer_ID
• product_ID
• product_rating
• time_of_review
• helpfulness (count of thumb-ups and thumb-downs received), identify the expert reviewers and derive the true rating of products.

### 1.3 Organization of the study

Section 2 introduces the previous related works, existing techniques, and discusses the limitations of those techniques. Section 3 proposes and discusses two new algorithms for detecting expert reviewers and determining the true rating of products. In section 4, we compare the effectiveness of the algorithms and performance of the experts suggested by our algorithms against the existing techniques with the help of three real world datasets of different categories of experiential products. We conclude our study in section 5 summarizing our work and pointing out the possible future research direction.

## 2 Existing techniques

All over the web, we find the following techniques employed for deriving the product-rating and highlighting the reviews considered coming from probable experts.

### 2.1 Secret Proprietary techniques

Amazon ranks reviewers based on their secret algorithm [14]. They state that rank of a reviewer is determined by overall helpfulness of all their reviews and their quantity of reviews. However, to ensure that the reviewers remain active, they include a penalty term that controls the contribution of a review to the reviewer's total score. The more recently a review is written, the greater is its impact on the rank. For product rating, amazon uses a machine-learning algorithm that uses the rating given by the reviewers, the ages of the reviews, whether the reviews are from verified purchasers, and some hidden factors that establish reviewer trustworthiness [15]. The problem with these Secret Proprietary techniques are-they are not open source. Internet Movie Database (IMDB) also has not published how they derive the true rating of a movie from the reviews.

### 2.2 Open source techniques

#### 2.2.1 Techniques for finding the true product rating

Apart from the proprietary techniques, we found the following open-source methods used in various websites, each of which has its limitation.

a) **Average using all reviews**

**Figure 1:**
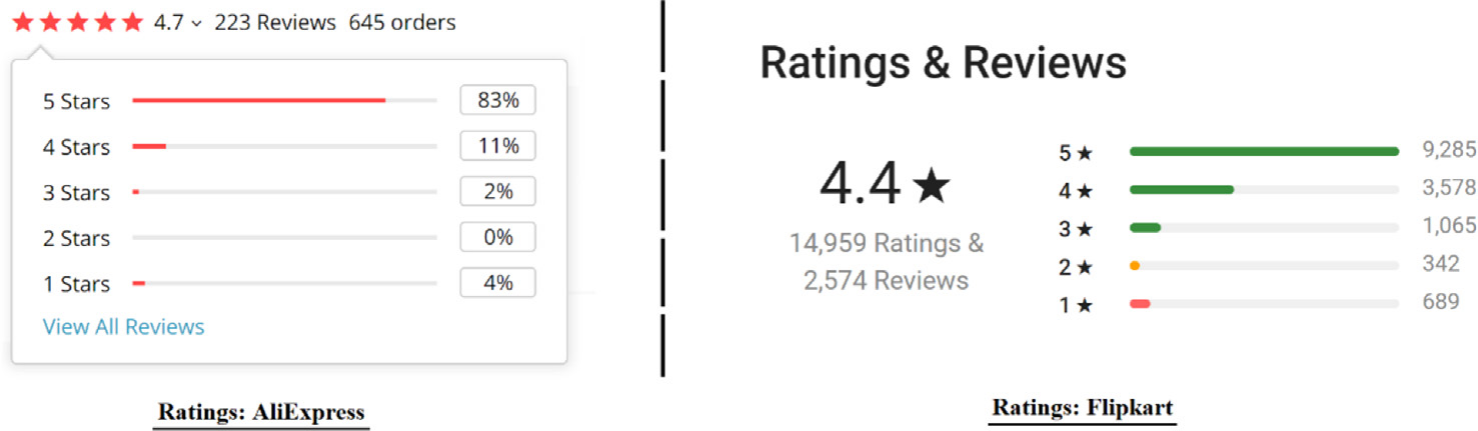
Example of avg. overall rating derived from reviews in aliexpress and flipcart.

This is the most widely used technique for deriving the product rating from reviews. E-commerce giants like AliExpress, Flipkart, Lazada, etc., use this technique (see Figure 1). For a product X, if there are *N* numbers of reviews, then the true product rating is approximated by using the following formula [16] given in Eq. (1):

(1) $$Rating_{X}=\frac{\sum_{i=1}^{N}ReviewRating_{i}}{N}$$

Eq. (1) works well when the number of reviews is large, but works poorly when the quantity of reviews is small.

b) **IMDB's “true Bayesian average”**

IMDB publishes weighted vote averages rather than the simple arithmetic mean to reduce attempts of ‘vote stuffing.’ They have not disclosed the exact methods applied but indicated that various filters are applied to the raw data. However, they have published the following formula in Eq. (2) which they used to calculate the weighted rating, *W*, of the top 250 films [17]:

(2) $$W=\;\frac{R.V+C.M}{V+M}$$

where *R* is the average for the movie as a number from 0 to 10 (mean_Rating), *C* is the mean vote across the whole database, *V* is the number of votes for the movie and *M* is the minimum votes required to be listed in the Top 250 movies.

If *M* is too small, then the ratings will have extreme variability, but if it is too large, then it will give too much weighting to the mean rating, *C*. So *M* should be reasonable (currently IMDB uses *M* = 25000). As more and more votes for a particular movie are added, the movie will eventually get to the true rating. However, this method is not used in general because it assumes an unusually large number of reviews for each product, which is often not available for most products.

#### 2.2.2 Open-source straightforward techniques for finding the expert reviewers

Most eCommerce websites provide users with the option to sort reviews based on helpfulness (thumb-ups count), ascending/descending order of their rating, and newness. Given a user with *n1* thumb-ups and *n2* thumb-downs, besides the proprietary technique, we usually see the following techniques used by the eCommerce websites to detect the top reviewers (experts).

**Popularity:** The most used method uses the total number of thumb-ups as the score of the reviewer. A reviewer is as good as the total number of his acquired thumb-ups. A problem with this technique is that it completely ignores the thumb-downs. For example, a reviewer with ‘1000’ thumb-ups but ‘500’ thumb-downs could be ranked higher than a user with ‘999’ thumb-ups but 100 thumb-downs.

**Helpfulness Difference:** The second method is to use the difference between the number of thumb-ups and the number of thumb-downs as the reviewer's score. Although it takes into consideration the thumb-downs, it introduces a problem of its own. For example, a user with 200 thumb-ups and 100 thumb-downs (difference: 100) will be ranked lower than a user with 2000 thumb-ups and 1800 thumb-downs (difference: 200). This does not seem right because the first user has twice the thumb-ups than thumb downs, while the other user has only slightly more thumb-ups than thumb-downs.

**Thumb-ups Ratio/Proportion:** This method rank users by the ratio of thumb-ups to the total number of thumb-ups and thumb-downs. It works well when all the reviewers have got a large number of votes. However, it doesn't work well when the numbers of votes are small. For example, a reviewer with 999 thumb-ups and 1 thumb-down (thumb-ups ratio = 0.999) is ranked lower than a reviewer with a single thumb-up but no thumb-downs (thumb-ups ratio = 1.0).

### 2.3 Link-analysis based techniques

There has been a long-standing interest in modeling expertise in social networks and communities using modified versions of link analysis algorithms such as PageRank and HITS. Zhang et al. [18] modified the PageRank algorithm and proposed an algorithm ExpertiseRank to find experts in the question and answer community. Their algorithm considers whom a person has answered (novice or an advanced user) and how many people a person has answered. The number of answers given by a user (*na*) and the number of questions asked by the user (*nq*) are combined in one score, *Z-score*, calculated using the following Eq. (3):

(3) $$Z_{score}\;=\;\frac{\left(na-nq\right)}{\sqrt{na+nq}}$$

A person with high *Z-score* is considered to have higher expertise than a person with low *Z-score* value. Their analysis over a dataset from the Java developer forum indicated that a simple measure such as *Z-score* outperforms complex graph based algorithms such as ExpertiseRank, PageRank, and HITS in the assessment of the expertise of the users.

Jurczyk et al. [19] showed that the HITS algorithm outperforms classical graph measures such as in-degree, out-degree, and centrality for the identification of experts in Question Answer Communities such as Yahoo! Answers. Guha et al. [20] and Ziegler [21] explored the spreading of trust and distrust in online social media. They believe that trust is a passing attribute in network relationships. Bian et al. [22] proposed a mutual reinforcement framework for ranking a group of entities in the Q&A community.

However, the experiential product reviewing mechanism differs significantly from the mechanism used in the communities mentioned above. In question-answering communities, the enquirer of the question gives direct feedback and chooses the best answer from the set of answers. This feature is especially helpful in finding the best answerer/experts. Moreover, although link-based and probabilistic approaches are successful in ranking entities on the graph, as per our literature review, we found no research that addresses ranking the entities when the underlying system is an explicit bipartite graph with only forward edges. No one review is selected as the best review here.

## 3 Proposed techniques

In 3.1 and 3.2, we have proposed two algorithms respectively that address the limitations of existing approaches. Algorithm 1 is based on a combination of the straightforward approaches. Algorithm 2 is based on the link-analytic mutual reinforcement technique by modifying the HITS algorithm.

### 3.1 Simple filter and sort

Algorithm 1 Expert reviewer detection by Simple Filtering and Sorting (SFS)

**Table:** 

Input: All the product reviews.
Output: A list of the required number of experts.
BEGIN
1. Consider only the reviewers who have contributed sufficient (at least more than a *minimum discard threshold*) numbers of reviews.
2. Sort the selected reviewers from step 1 by their total *thumb-ups to thumb-down* differences. Select the top *N1* reviewers (or those whose differences are above certain threshold) from the sorted list. (The next two steps, steps 3 and 4, can be applied by altering their current order.)
3. Sort the remaining reviewers from step 2 by their helpfulness-proportion. Select the top *N2* reviewers (or those whose helpfulness-proportion are above a certain percentage) from the sorted list.
4. Sort the remaining reviewers from step 3 by their respective Mean Squared Error (MSE). Select the top required-number of reviewers (or those whose MSE are below a certain threshold) from the sorted list. These reviewers are the experts.
END

The first step of the Simple Filtering and Sorting (SFS) algorithm ensures that reviewers with a count of reviews less than minimum discard threshold are not considered in the process. Less than this threshold number of reviews is unlikely to be fruitful to capture the reviewers’ expertise. We can interpret this as a measure of imposing commitment towards the reviewing task as well.

Helpfulness vote is one of the most important factors in determining expertise. In step 2, we used helpfulness-difference. Prior research showed that a reviewer's reputation is positively associated with the number of helpful votes received [23, 24]. So the amount of helpfulness vote the reviews obtained indirectly captures a measure of expertise of the reviewer. Furthermore, it significantly influences the customer's purchase decision [25, 26, 27]. Past helpfulness records are positively associated with future helpfulness ratings obtained by a reviewer [28]. We did consider filtering by helpfulness-ratio in step 2, but the problem with filtering by helpfulness-ratio this early in step 2 is that it’s very likely to select those reviewers who have low “total accumulated thumb-ups” and zero or very few “total accumulate thumb-downs”. For example, reviewers with merely 1 thumb-up and 0 thumb-down have a perfect helpfulness-ratio of 1. This kind of non-expert reviewers is common in the community. So to avoid this problem, in step 2, we select the users based on helpfulness-difference.

The order of execution of the next two steps (steps 3 and 4) reflects which quality of the reviewers we give preference to for being considered as an expert: their accuracy or the clarity of response. Less MSE reflects the reviewer's ability to predict the true product quality. On the other hand, larger helpfulness proportion confirms the reviewer's ability to efficiently and clearly express his domain knowledge. Helpfulness proportion also indirectly measures the domain knowledge of the reviewer. Another important point to notice is: since we are using MSE as one of the main metrics to determine expertise, a corrupt user can guess the product rating from the earlier reviews and post a perfect rating later resulting in minimal MSE. But since early written reviews receive longer exposure, they usually get more votes than the reviews with similar quality posted later. So step 2 is crucial to discard this type of corrupt reviewer.

To perform step-4, we need to calculate the Mean Square Error (MSE). MSE measures the average of the squares of the “errors” of an estimator. If ***Ỹ*** is a vector of ***n*** predictions, and ***Y*** is the vector of the true values, then the (estimated) MSE of the predictor is calculated using the following Eq. (4):

(4) $$MSE=\frac{1}{n}\sum_{i=1}^{n}{\left({\tilde{\text{Y}}}_{i}-Y_{i}\right)}^{2}.$$

To calculate the MSE of a reviewer, we need a way to calculate the ‘True Rating’ of every product that the reviewer reviewed. Two different techniques have been used to derive the true ratings from reviews. The first one is the existing “average of all the ratings given by the reviewers” (Eq. (1) used by aliexpress, flipcart etc.). The second one, we propose now, takes into account the impact of helpfulness. The equation for the second technique is given below (Eq. (5)).

• **True rating as the average of all review-ratings considering helpfulness votes**

When we take into consideration the helpfulness of the reviews and consider each thumb-up as a boosting of the same review, then for a product X, if there are *N* numbers of reviews, the true rating is approximated by-

(5) $$Rating_{X}=\frac{\sum_{i=1}^{N}\left\{Review\;Rating_{i}\times \left(1+ThumbUp_{i}\right)\right\}}{N+\sum_{i=1}^{N}ThumbUp_{i}}$$

In Eq. (5), those who find a review helpful and contributes a thumb-up is considered as giving the same rating. So if a review gets 100 thumb-ups, it is treated like 101 individual reviews.

### 3.2 Expert detection by link-analysis

**Figure 2:**
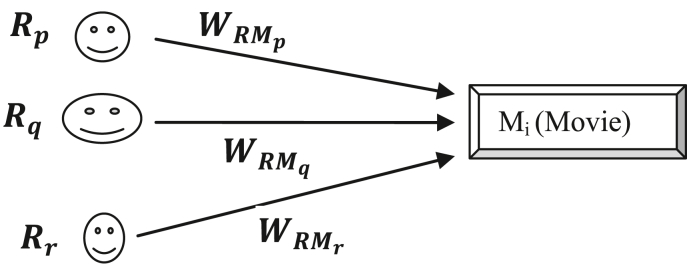
Movie's true rating and weight as a function of reviewer's weight, reviewer's given rating, and the number of reviews.

We can view the collection of reviews as a directed graph *G* and denote it as: $G\left(R,M,\;W_{RM},W_{MR}\right)\;$where *R* is the set of reviewers, *M* is the set of movies (products), $W_{RM}$ is the set of directed weighted edge from reviewers to movies, $W_{MR}$ is the set of directed weighted edge from movies to reviewers. We note that not all reviewers should impact the movie ratings equally. The reviews of the experts and the reviews which have more helpful votes should play a greater role than other reviews. So the weights are updated according to the reviewer's expertise level and the helpfulness of the review. Figure 2 depicts the process.

**Figure 3:**
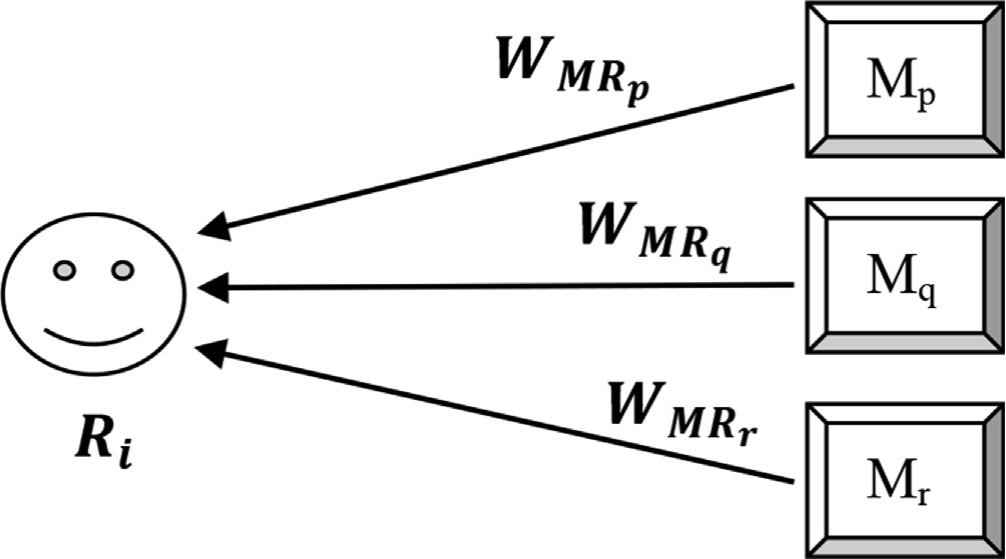
Reviewer's weight (Expertise_score) as a function of movie-weight, deviation from the true rating, and the number of reviews.

On the other hand, not all movies should play an equal role in determining experts. A movie with a large number of reviews should be given a higher weight (i.e., the deviation of the reviewer-given rating from the true-rating of the movie should have greater impact). Figure 3 depicts the process.

Now we propose the Algorithm 2 considering these facts. The algorithm is based on the popular link analyzer algorithm HITS [29].

Algorithm 2 Expert Reviewer detection by mutual reinforcement.

**Table:** 

Input: All the product reviews.
Output: A list of the required number of experts.
BEGIN
1. Retrieve a large enough root set of potential experts from the reviewers.
2. Assign weights to products by their respective number of reviews.
3. Sort the products by the earliest posting time of their associated reviews.
4. REPEAT until all products are processed 4.1 Read all the reviews of the next product from the sorted list. 4.2 Determine the true rating of the product from the (weighted) reviews. 4.3 Calculate the reviewers' weighted-average-error. 4.4 Update reviewers' weight.
5. Calculate the score of the reviewers. Select the top required number of experts from them and then sort them by their MSE
END

In step-1, for example, if we want to get the top 1% reviewers, we may begin by constructing a root set of potential experts by selecting the 15–20% reviewers who have the largest thumb-ups to thumb-downs difference from the set of all reviewers each of whom have made 4–5 times more reviews than the population average. This step will ensure the potential expert reviewer's commitment to the community. Step-2 is very subjective and depends on the problem domain of the particular experiential product category. For example, we may assign the top 10% item with most reviews a weightage of 10, and the next 10% could be assigned a weightage of 9 and so on. Alternatively, we may assign items with a certain number of reviews a weightage of 10, and so on. The algorithm contains three important calculations in the REPEAT section (step-4):

1. Determine the true rating of the product from the (weighted) reviews. (step-4.2)
2. Calculate the reviewers' weighted-average-error. (step-4.3)
3. Update reviewers' weight. (step-4.4)

We determine the (weighted) rating of the item (in Step 4.2) using the following formula in Eq. (6). It is similar to the formula used in Eq. (5) except that, in Eq. (6), reviewers are assigned weight ($UW_{user_{i}}$) which influence the true rating of the product.

(6) $$RT_{item_{X}}=\frac{\sum_{i=1}^{N}\;UW_{user_{i}}\times User\;Given\;Rating_{user_{i},item_{x}}\times \left(1+TUC_{user_{i},item_{x}}\right)\;}{\sum_{i=1}^{N}\left\{UW_{user_{i}}*\left(1+TUC_{user_{i},item_{x}}\right)\;\right\}}$$

where *N* is the number of reviewers of that movie, $RT_{item_{X}}$ is the weighted rating of $item_{x},\;UW_{user_{i}}\;$is *user*_*i*,_’s weight (can be assigned in various ways; one such way is described in 4.2.3.), $TUC_{user_{i},item_{x}}$ is the thumb-up count of $user_{i}$’s review on $item_{x}$, and $UserGivenRating_{user_{i},item_{x}}$is $user_{i}$’s given rating to $item_{x}$ .

We update the weighted-average-error in step-4.3 using the following formula in Eq. (7):

(7) $$New\_Weighted\_Avg\_Error_{User_{i}}=\frac{Total\_Old\_Error+\left(Error\times Item\;Weight_{item_{x}}\right)}{New\_Weighted\_Total\_Item\_Count_{user_{i}}\;}$$

where $Total\_Old\_Error=\left\{Old\_\_Weighted\_Avg\_error_{User_{i}}\times Old\_Weighted\_Total\_Item\_Count_{user_{i}}\right\}$, $Error=\left\{\left|UserGivenRating_{user_{i},item_{x}}-RT_{item_{X}}\right|\right\}$, ${\text{Item Weight}}_{{\text{item}}_{\text{x}}}$ is ${\text{item}}_{\text{x}}$’s weight *(*can be assigned in various ways; one such way is described in 4.2.3.) and. $New\_Weighted\_Total\_Item\_Count_{user_{i}}=\left\{\;Old\_Weighted\_Total\_Item\_Count_{user_{i}}+ItemWeight_{item_{X}}\right\}.$

In step-4.4, we update the weight of the reviewers (who reviewed this item) using their respective weighted-average-error. A reviewer's weight is inversely proportional to his weighted-average-error. Larger weighted-average-error causes the assignment of less weight to the user. However, this step is subjective to the domain of application because the range of the weighted-average-error changes according to the domain of the application. For example: in a particular domain where reviewers rate products in a rating scale of 1–5, we might assign weight 1 to those who have weighted-average-error > 1.0, weight 2 to those who have weighted-average-error between 0.8 to 1.0, weight 5 to those who have weighted-average-error < 0.2 and so on. On another scale, we might assign weight 5 to those with weighted-average-error < 5.

At the last step, we calculate the score $S_{i}$ of $Reviewer_{\text{i}}$ using the following formula presented in Eq. (8):

(8) $$S_{i}=\left(E_{i}.\frac{P_{i}}{P_{i}+M}\;+\;C.\;\frac{M}{P_{i}+M}\right)$$

where $E_{i}\;$is the weighted average error of $Reviewer_{i}$, *C* is the mean of weighted average error on the reviewers' set.$P_{i}$ is the weighted_total_item_count of $Reviewer_{i}$, and *M* is an influence-Constant. Smaller value of $S_{i}$ indicates higher confidence on the expertise level of the reviewer. Eq. (8) ensures that reviewers with a sufficiently large number of reviews get selected as the experts even though reviewers with little (threshold) reviews may have a slightly lower weighted average error. For example, we might want to ensure that a reviewer with 1000 reviews with weighted-average-error of 0.33 gets ahead than a reviewer with 50 reviews with weighted-average-error of 0.30. A carefully selected value of *M*, the influence-constant, helps to achieve this goal. The value of the influence constant (*M*) cannot be too low or too high. It takes a similar kind of role it played in the formula provided by IMDB in Eq. (2). If we select too low a value for *M*, then reviewers with less weighted-average-error but with few reviews will be selected as experts. We do not want that. On the other hand, if we select a too high value for *M*, then only the reviewers with a large number of reviews will be selected, even if their weighted average-errors are not small enough to be an expert. We want an intermediate value of *M* between these two extremes. So we have to be careful in selecting the value of *M*. In the next section, we evaluated the performance of the two proposed algorithms on three datasets collected from Amazon.com.

## 4 Evaluation

Since there is no ground truth on the true rating of the products, and nor on the reviewers, deciding how to evaluate the proposed models is another interesting problem. We cannot exactly answer the question, “Can we rank reviewers correctly, according to expertise?” Therefore, we propose an approximation– to answer a related question: “After estimating the true ratings of the products, how well do the algorithms perform to detect expert reviewers and minimize the cost function?”

To answer this, we have to do three things:

1. Estimating the true rating of the products based on user ratings (reviews).
2. Finding the experts using our proposed approaches.
3. Comparing the performance against the traditional approaches and against each other.

### 4.1 The datasets

Three datasets from Amazon.com have been used in this study. Amazon is one of the largest online retailers. Also, it has one of the most active reviewing communities online. Amazon's product review consists of the following: the product id, the reviewer id, the review body, the time of the posting of the review, a helpfulness count (thumb-ups/total), and a rating of the product on a discrete 5-point scale. The first dataset for this study was collected from Stanford University's “Stanford Network Analysis Project” [30]. It consists of movie reviews. The second and third dataset is collected from the ‘University of California, San Diego's recommender systems datasets [31]. The second dataset contains reviews of Digital Music products, and the third dataset contains reviews from Amazon Instant Video. The three datasets contain 7911684, 836006, and 583933 reviews, respectively. More information about the datasets is given in their respective experimentation subsections.

### 4.2 Experimentation and result: dataset 1

The data span a period of more than 10 years up to October 2012. There are in total 889176 reviewers, 253059 movies, and almost eight million reviews on the dataset. In this dataset, among the 889176 reviewers, 16341 reviewers have contributed more than 50 reviews each. We limit out search for experts among these reviewers. Our goal is to find 200 experts from them, which is roughly 1.2% of the eligible reviewers (with >50 reviews).

#### 4.2.1 Performance of different straightforward techniques and SFS algorithm

**Figure 4:**
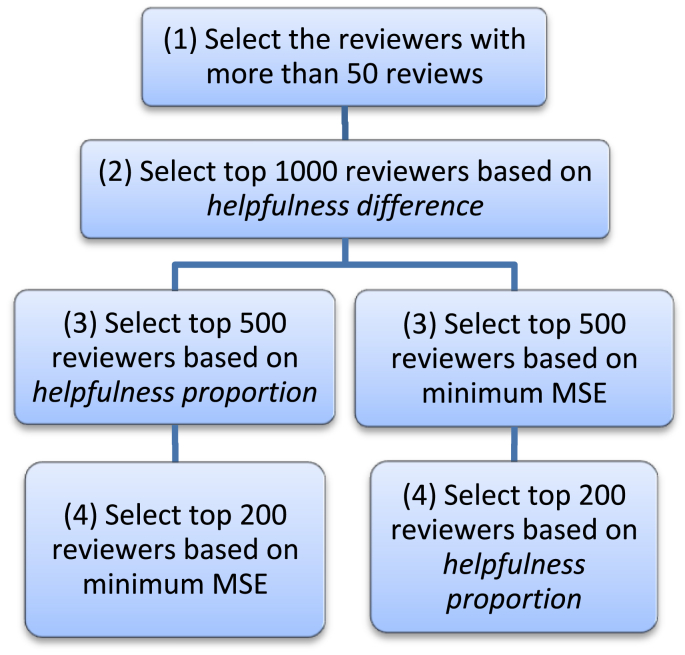
Customization of SFS algorithm for dataset 1.

For dataset 1, the SFS algorithm (Algorithm 1) is customized as follows (see Figure 4):

In step 2 of this customized algorithm, 1000 potential candidates were selected, which is roughly 6% of the eligible population. Then in step 3, 50% of the potential candidates were retained and the other 50% discarded; which leaves us with roughly 3% of the eligible population. Finally, in step 4, we narrowed down to the desired 1.2% of reviewers as experts suggested by this algorithm.

**Figure 5:**
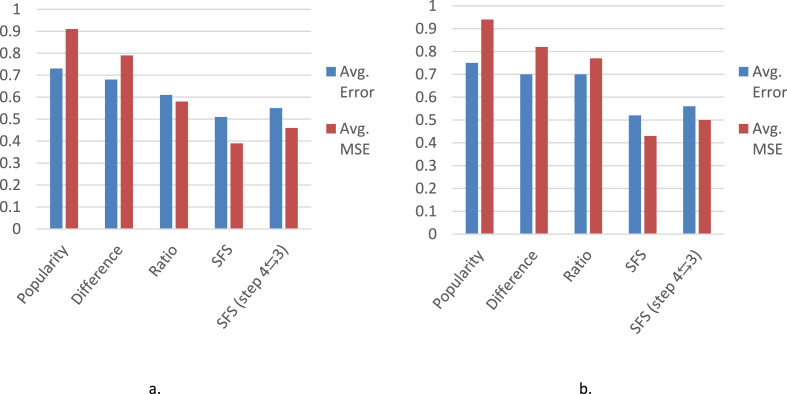
Avg. error and avg. MSE of the reviewers suggested by different expert reviewer detection techniques. a. True Rating considering only review-rating. b. True Rating considering helpulness votes.

Table 1 and the column-charts in Figure 5 show that our simple filter and sort (SFS) technique outperforms the usual straightforward methods. When the product rating is calculated without considering the helpfulness votes, its recommended experts achieved almost 33% {(0.585−0.391)/(0.585)} reduction in MSE and 15% {(0.610−0.517)/(0.610)}reduction in average error than the best of the straightforward techniques. The SFS algorithm outperformed the usual straightforward methods even when the product rating is calculated considering the helpfulness votes. This time, the experts from this algorithm achieved almost **43%** {(0.766−0.433)/(0.766)} reduction in MSE and **25%** {(0.704−0.524)/(0.704)} reduction in average-error.

**Table 1:** Performance of the different expert reviewer detection techniques.

Method of expert reviewer detection	Product's True Rating considering only review-rating (Eq. (1))				Product's True Rating considering helpfulness votes (Eq. (5))			
	No. of reviews made	No. of movies covered	Avg. Error	Avg. MSE	No. of reviews made	No. of movies covered	Avg. Error	Avg. MSE
Popularity	447827	96840	0.697	0.823	447827	96840	0.718	0.854
Difference	407101	94764	0.684	0.790	407101	94764	0.704	0.817
Help-ratio	14165	10311	0.610	0.585	14165	10311	0.704	0.766
SFS	119176	54621	0.517	0.391	115454	57516	0.524	0.433
SFS (step 4⇆3)	108196	55508	0.550	0.456	110441	57874	0.555	0.499

#### 4.2.2 Expert reviewer detection by mutual reinforcement

In step-1 of our experiment, we construct the root set of potential experts. We experimented with two different root sets. Set-1 was created by selecting all reviewers, each of whom has made more than 50 reviews. There were 16341 reviewers in set-1. Set-2 contained the top 2000 reviewers who have the largest thumb-ups to the thumb-downs difference from set-1.

**Figure 6:**
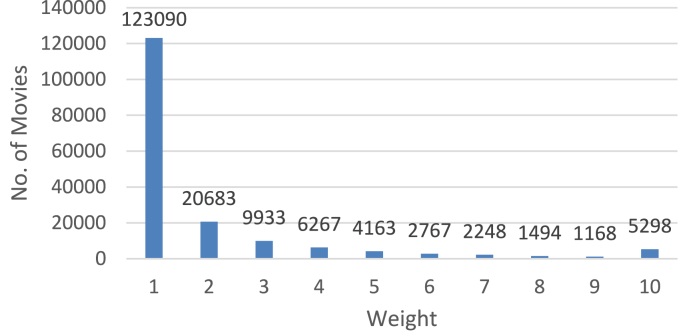
Distribution of Movies according to weight.

In step-2 of this algorithm, we assign weights to movies according to the number of reviews. Since weights assigned to movies will impact the reviewer's weight (if there's a deviation of reviewer's given rating from the true rating), how we assign weights to movies is of great importance. In our experiment, we assigned ‘1’ as the weight to those movies with 1–30 reviews. We assigned weight ‘2’ to those movies with 31–60 reviews inclusive, and so on for each increment by 30 in the review-count range. Finally, we assigned weight ‘10’ to those movies with more than ‘270’ reviews. We believe the derived rating of those movies of weight ‘10’ can be considered the true rating with full confidence. The weight distribution of the associated movies is shown in Figure 6.

**Figure 7:**
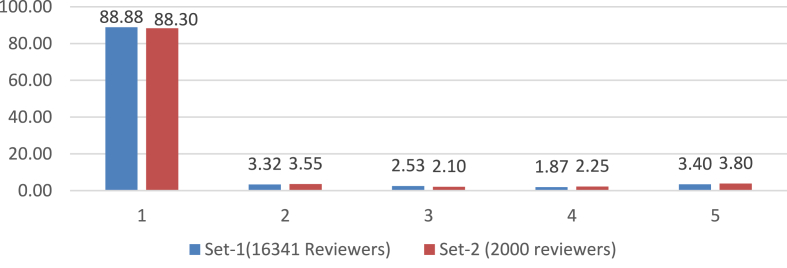
Percentage Distribution of reviewers according to their weight in the two root sets.

Another similarly important step (step 4.4) is to assign and update reviewers' weight. This procedure is also subjective and depends on the problem domain. We updated the reviewer's weight based on the reviewer's current average error. We experimented with several distributions of weight and finally used the criteria mentioned in Table 2 for updating the user's weight. The resulted percentage distribution of all the reviewers from the two root sets according to their respective final weight is given in Figure 7. Understandably most of the reviewers are of the lowest weight (i.e., 1).

**Table 2:** Reviewer's weight according to his Avg. error.

Avg. Error	<0.35	~ <0.40	~ <0.45	~ <0.50	≥0.50
Weight	5	4	3	2	1

The final step of the algorithm involves calculating the score of the reviewers from their weighted avg. error and their weighted number of reviews (using Eq. (8)). The value of the influence-Constant, *M,* could be interpreted as the minimum number of reviews needed to consider a reviewer as a potential expert. We would want *M* to be sufficiently large so that we can be sure that the identified experts have contributed quite a lot of reviews. At the same time, for experts, *M* should be much smaller than *weighted_total_item_count* of the reviewer so that it renders the effect of the population's weighted average error on score insignificant. We have performed several trials for various values of *M* to find the 200 experts. The following two tables, Tables 3 and 4, present the performance of 30 different sets of potential experts obtained by varying the value of *M* on the two root sets of reviewers. Table 3 presents the avg. error, avg. MSE, coverage of movies, and the quantity of reviews made by each of the 30 set of experts. Table 4 compares the avg. error and avg. MSE of each of the 30 set of experts for the three different true-rating estimation techniques.

**Table 3:** Effect of the influence-constant, *M*, on reviewers’ avg. error, avg. MSE, movie coverage and count of reviews.

Value of *M*	When experts were derived considering set-1 (Root set: 16341 Reviewers)				When experts were derived considering set-2 (Root set: 2000 Reviewers)			
	No. of reviews made	No. of movies covered	Avg. Error	Avg. MSE	No. of reviews made	No. of movies covered	Avg. Error	Avg. MSE
100	19298	11322	0.275	0.153	55555	33388	0.397	0.300
200	24886	14609	0.288	0.163	64972	37555	0.407	0.312
300	40550	23457	0.310	0.190	78397	41877	0.414	0.323
400	58792	32688	0.334	0.221	87544	44202	0.423	0.336
500	75927	38524	0.361	0.253	105632	49793	0.428	0.343
600	109930	48157	0.390	0.289	137092	57175	0.439	0.356
700	139423	55477	0.412	0.318	146653	58577	0.443	0.360
800	157614	57932	0.429	0.341	153345	59376	0.453	0.370
900	177250	61291	0.454	0.373	159047	60421	0.457	0.374
1000	200893	66269	0.466	0.390	163826	60740	0.461	0.376
1500	251639	71879	0.506	0.438	211483	68229	0.482	0.404
2000	299274	77785	0.529	0.471	227989	70317	0.498	0.427
3000	323883	80025	0.543	0.494	256831	73435	0.520	0.457
5000	366149	83063	0.563	0.527	294411	78051	0.538	0.486
10000	420275	87878	0.581	0.560	312585	79834	0.552	0.507

**Table 4:** Avg. error and avg. MSE of several sets of expert reviewers (due to different M) for different true-rating derivation technique.

Value of *M*	Using set-1 (Root set of 16341 Reviewers)						Using set-2 (Root set of 2000 Reviewers)					
	Technique for deriving true product rating						Technique for deriving true product rating					
	Mutual reinforcement		Only review rating		Considering review helpfulness		Mutual reinforcement		Only review rating		Considering review helpfulness	
	Avg. Error	Avg. MSE	Avg. Error	Avg. MSE	Avg. Error	Avg. MSE	Avg. Error	Avg. MSE	Avg. Error	Avg. MSE	Avg. Error	Avg. MSE
100	0.275	0.153	0.391	0.241	0.372	0.224	0.397	0.300	0.500	0.413	0.477	0.389
200	0.288	0.163	0.392	0.237	0.384	0.236	0.407	0.312	0.504	0.417	0.485	0.398
300	0.310	0.190	0.403	0.253	0.400	0.260	0.414	0.323	0.504	0.418	0.488	0.405
400	0.334	0.221	0.413	0.267	0.417	0.289	0.423	0.336	0.506	0.423	0.495	0.416
500	0.361	0.253	0.425	0.281	0.436	0.315	0.428	0.343	0.506	0.421	0.498	0.419
600	0.390	0.289	0.442	0.310	0.458	0.348	0.439	0.356	0.504	0.420	0.503	0.427
700	0.412	0.318	0.454	0.328	0.475	0.374	0.443	0.360	0.506	0.421	0.505	0.428
800	0.429	0.341	0.464	0.338	0.489	0.394	0.453	0.370	0.507	0.422	0.512	0.435
900	0.454	0.373	0.473	0.356	0.505	0.421	0.457	0.374	0.507	0.419	0.515	0.438
1000	0.466	0.390	0.479	0.367	0.514	0.436	0.461	0.376	0.508	0.417	0.517	0.438
1500	0.506	0.438	0.504	0.391	0.544	0.474	0.482	0.404	0.514	0.422	0.530	0.458
2000	0.529	0.471	0.517	0.413	0.562	0.504	0.498	0.427	0.520	0.431	0.542	0.476
3000	0.543	0.494	0.527	0.429	0.575	0.526	0.520	0.457	0.524	0.431	0.556	0.497
5000	0.563	0.527	0.542	0.456	0.591	0.554	0.538	0.486	0.532	0.439	0.569	0.518
10000	0.581	0.560	0.554	0.481	0.606	0.583	0.552	0.507	0.535	0.448	0.577	0.533

When we consider the coverage of movies by the expert reviewers suggested by the SFS and SFS(step 4⇆3) algorithms in Table 1, we observe that they covered 54–58 thousand movies and the number of reviews made by the expert reviewers is between 110 to 119 thousand. Table 3 shows that when *M* is set to 700 and 800 for set-1 of potential experts, the recommended 200 experts by this *mutual reinforcement technique* covers 55477 and 57932 movies respectively and made 139423 and 157614 reviews respectively. On the other hand, when *M* is set to 600 and 700 for set-2, the recommended 200 experts covered 57175 and 58577 movies and made 137092 and 146653 reviews respectively.

In case of performance metrics (the avg. error and avg. MSE), we observe in Table 1 that the lowest avg. error and avg. MSE among the straightforward techniques, SFS and SFS(step 4⇆3) algorithms was obtained by the SFS algorithm. When true product ratings are derived considering only review-ratings, the lowest avg. error and avg. MSE are 0.517 and 0.391 respectively. When true product ratings are derived considering the helpfulness votes of the review, the lowest avg. error and avg. MSE are 0.524 and 0.433 respectively.

In Table 5, we see that all four sets of reviewers recommended by the mutual reinforcement technique caused less average error than the recommended reviewers by SFS. The best among these are from root set-1 with *M = 700*. Compared to the SFS algorithm, the avg. error and avg. MSE was reduced by 12.15% and 15.87%, 12.19% and 16.11%, and 9.35% and 13.63% respectively when product's true rating is estimated by mutual reinforcement, an average of review ratings, and weighted average of review ratings considering helpfulness votes respectively. That is a significant performance improvement reducing both avg. error and avg. MSE. This set of reviewers covered 55477 movies and made more reviews than the recommended reviewers by SFS. It is also evident from Table 5 that among the three true product rating derivation techniques, the mutual reinforcement technique causes the least avg. error for a set of experts irrespective of the expert-reviewer finding algorithms, which indicates that this product rating is closest to reflect the opinion of the expert reviewers.

**Table 5:** Performance comparison between experts of SFS algorithm and different candidates from *mutual reinforcement* algorithm.

Method of expert reviewer detection			True product rating using mutual reinforcement		True product rating using only review ratings		True product rating Considering review helpfulness	
			Avg. Error	Avg. MSE	Avg. Error	Avg. MSE	Avg. Error	Avg. MSE
SFS			0.469	0.378	0.517	0.391	0.524	0.433
Mutual reinforcement	Root set-1	*M* = 700	0.412	0.318	0.454	0.328	0.475	0.374
		*M* = 800	0.429	0.341	0.464	0.338	0.489	0.394
	Root set-2	*M* = 600	0.439	0.356	0.504	0.420	0.503	0.427
		*M* = 700	0.443	0.360	0.506	0.421	0.505	0.428

When we studied Table 5, we observed an interesting phenomenon about the performance of experts from root set-2 when avg. error and avg. MSE were calculated using only review ratings. In this particular case, the avg. error showed a reduction compared to the SFS algorithm, but there is an increase in the avg. MSE. It prompted us to examine the set of reviewers. Figure 7 presents the percentage distribution of reviewers by their weight for the two root sets. In the cases of reviewers set with *M = 600* and *M = 700* in root set-2, we notice that, in both cases, there are significantly more reviewers with weight-1 than with weight-5. Since less weight indicates more distance from true rating (e.g., Table 2), their squares contribute more to the Mean Squared Error.

**Figure 8:**
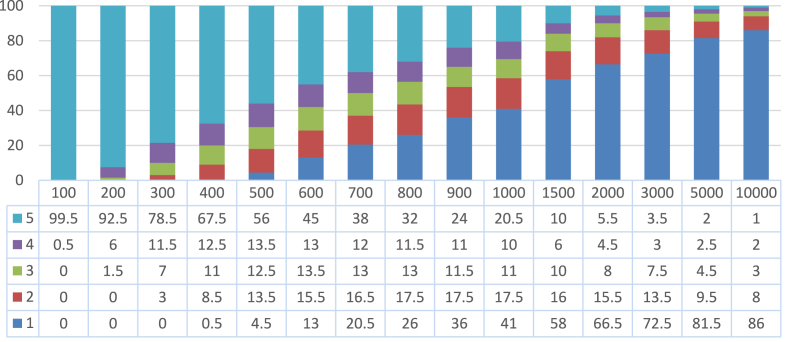
Percentage distribution of the various sets (different *M*) of 200 recommended reviewers from root set-1 by their weight.

**Figure 9:**
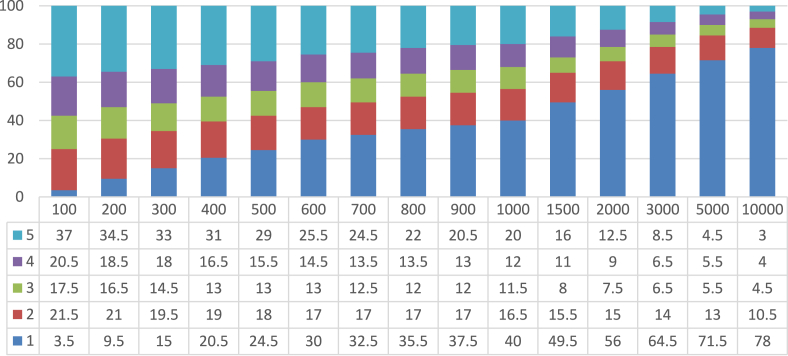
Percentage distribution of the various sets (different *M*) of 200 recommended reviewers from root set-2 by their weight.

From Table 3 and Figures 8 and 9, we observe the impact of *M* as expected; lower values of *M* produce sets of experts with lower coverage of movies and review-count, but with less avg. error. On the other hand, higher values of *M* produce sets of experts with higher coverage of movies and number of reviews, but with greater avg. error.

### 4.3 Experimentation and result: dataset 2

This dataset is much smaller and contains reviews of Digital Music products from Amazon. The data span a period of more than 16 years up to July 2014. There are, in total, 266414 reviewers and 836006 reviews on the dataset. Among its 478235 products, 119620 have more than one reviews. In this smaller dataset, 2314 reviewers have contributed more than 40 reviews each. This additional initial subset of potential expert reviewers was created by lowering the initial threshold of 50 reviews from the previous experiment to 40 reviews for this smaller dataset because, even after lowering the threshold to 40 reviews, only 2314 reviewers were left (compared to over 16 thousand in the dataset 1). We limit our search for experts among these reviewers. Our goal is to find 50 experts from them, which is roughly 2.2% of the eligible reviewers (with >40 reviews).

#### 4.3.1 Performance of different straightforward techniques and SFS algorithm

**Figure 10:**
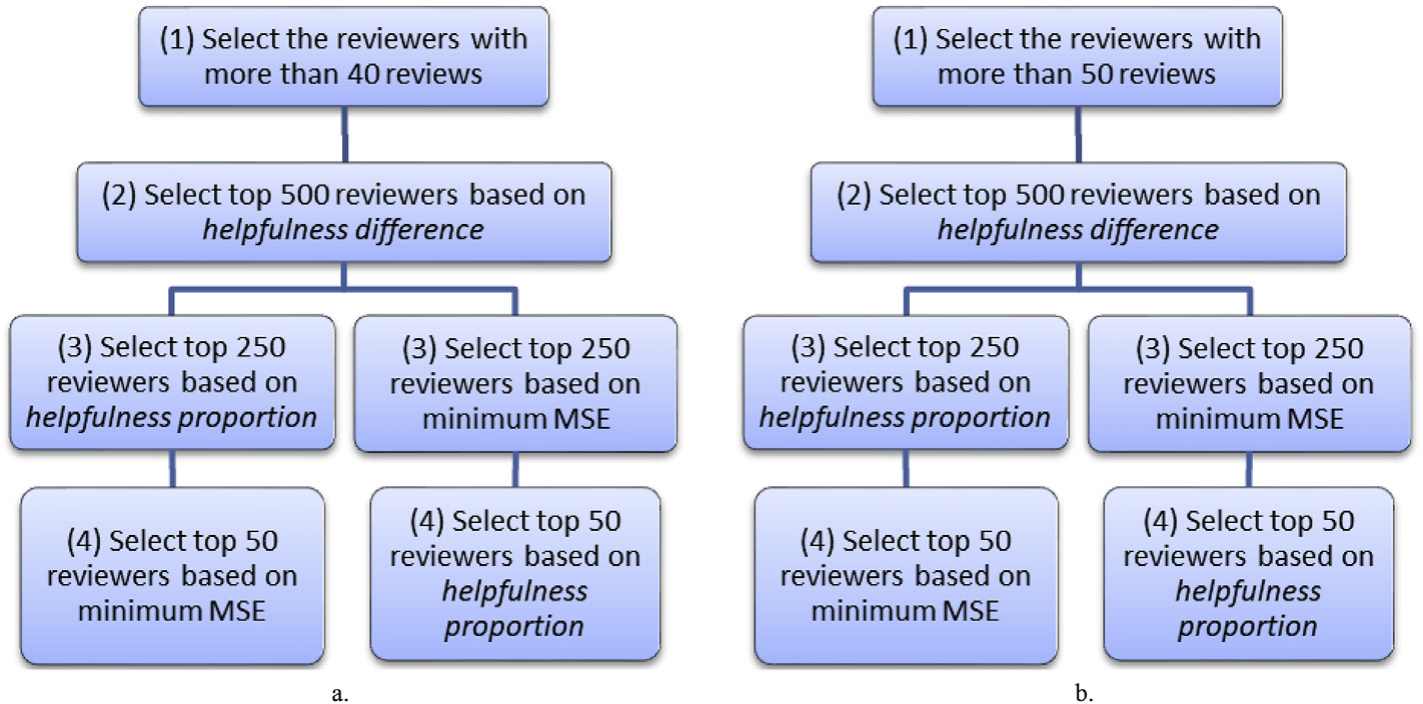
Customization of SFS algorithm for dataset 2. a. Initial set consisting of reviewers with >40 reviews. b. Initial set consisting of reviewers with >50 reviews.

For dataset 2, the SFS algorithm has been experimented with two different eligibility-threshold for creating the initial set of potential expert reviewers. Both customizations of the SFS algorithm are illustrated in Figure 10.

As seen on Figure 10, an additional initial set was created with reviewers reviews to explore the impact of varying the eligibility threshold. In step 2 of this customized algorithm, we selected 500 potential candidates, which is roughly 21.6% of the eligible population. Then in step 3, we retain 50% of the potential candidates and discard the other 50%, which leaves us roughly 10.8% of the eligible population. Finally, in step 4, we narrowed down to the desired 2.2% of experts suggested by this algorithm.

**Figure 11:**
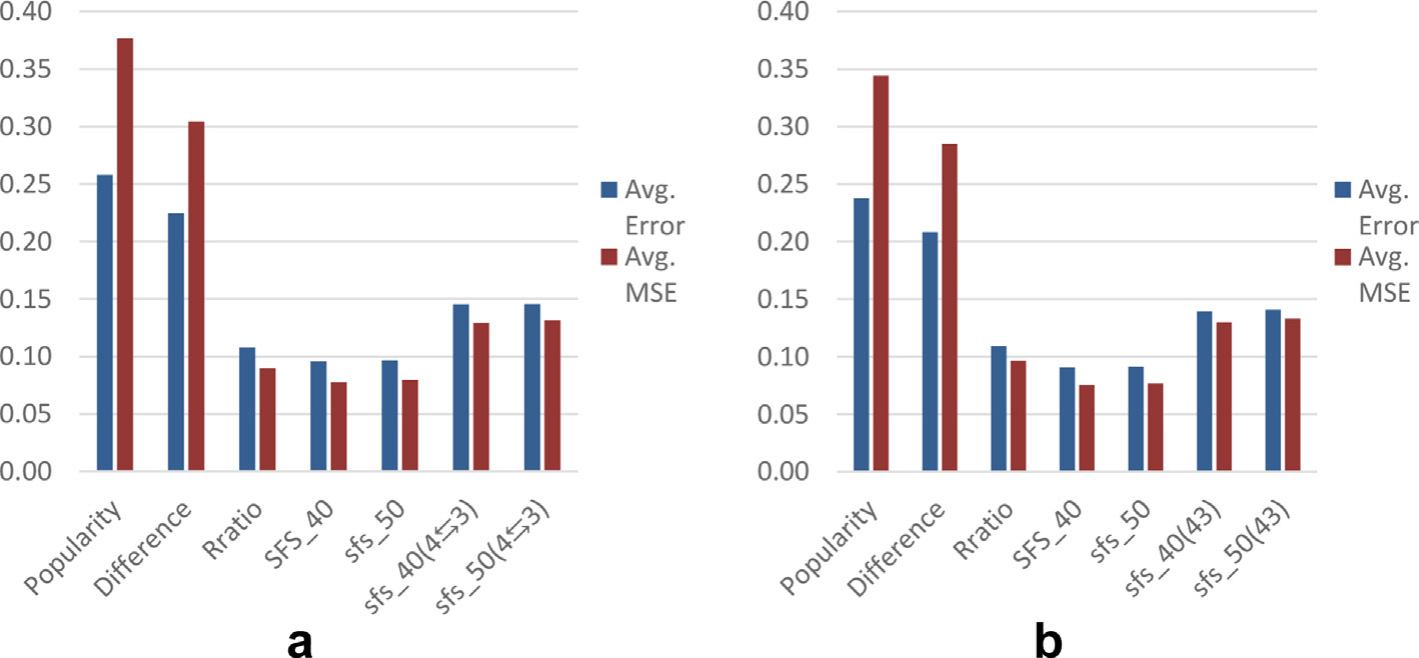
Avg. error and avg. MSE of the different expert reviewer detection techniques. a. True Rating considering only review-rating. b. True Rating considering helpulness votes.

Table 6 and the column-charts in Figure 11 show that the SFS technique continues to outperform the usual straightforward methods. When the product rating is calculated considering only the review-rating, its recommended experts achieved almost 11% {(0.090−0.080)/(0.090)} reduction in MSE and 10% {(0.108−0.097)/(0.108)}reduction in average error than the best straightforward technique. When the product rating is calculated considering the helpfulness votes, the experts achieved almost **20**% {(0.097–0.077)/(0.097)} reduction in MSE and **15**% {(0.109–0.092)/(0.109)} reduction in average-error.

**Table 6:** Performance of the different expert reviewer detection techniques.

Method of expert reviewer detection	Product's True Rating considering only review-rating (Eq. (1))				Product's True Rating considering helpfulness votes (Eq. (5))			
	No. of reviews made	No. of products covered	Avg. Error	Avg. MSE	No. of reviews made	No. of products covered	Avg. Error	Avg. MSE
Popularity	35060	31926	0.258	0.377	35060	31926	0.238	0.344
Difference	25689	23821	0.225	0.304	25689	23821	0.208	0.285
Help-ratio	3166	3073	0.108	0.090	3166	3073	0.109	0.097
SFS_40	8765	8627	0.096	0.078	7752	7631	0.091	0.076
SFS_50	9144	8999	0.097	0.080	8551	8424	0.092	0.077
SFS_40 (step 4⇆3)	4528	4422	0.145	0.129	4554	4438	0.139	0.130
SFS_50 (step 4⇆3)	5133	4997	0.146	0.132	5073	4931	0.141	0.133

#### 4.3.2 Expert reviewer detection by mutual reinforcement

Similar to the previous experiment, we constructed two root sets of potential experts. Set-1 was created by selecting those reviewers each of whom has made more than 40 reviews. We lowered the initial threshold of 50 reviews to 40 because even after lowering, there were 2314 reviewers in set-1. We created set-2 of potential experts by selecting the top 1000 reviewers who have the largest thumb-ups to the thumb-downs difference from the community. There are 197621 digital music products associated with the set-1 reviewers and 109756 products associated with set-2 of reviewers. However, the smallness of the dataset is revealed by the fact that almost 70% of the associated products in both sets have only a single review each.

**Figure 12:**
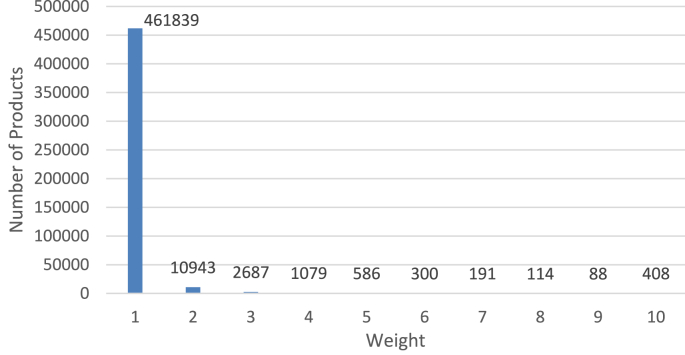
Distribution of digital music products according to weight.

In step-2 of this algorithm, we assign weights to products according to the number of reviews. Because this dataset is smaller in terms of reviews, we changed the thresholds for the weight categories. In our experiment, we assigned ‘1’ as the weight to the products with 1–5 reviews (inclusive). We assigned weight ‘2’ to the products with 6–10 reviews (inclusive), and so on for each increment by 5 in the review-count range. Finally, we assigned weight ‘10’ to products with more than ‘45’ reviews. The higher weight of a product is deemed to signify stronger confidence in its true-rating. The weight distribution of all the music products is shown in Figure 12.

**Figure 13:**
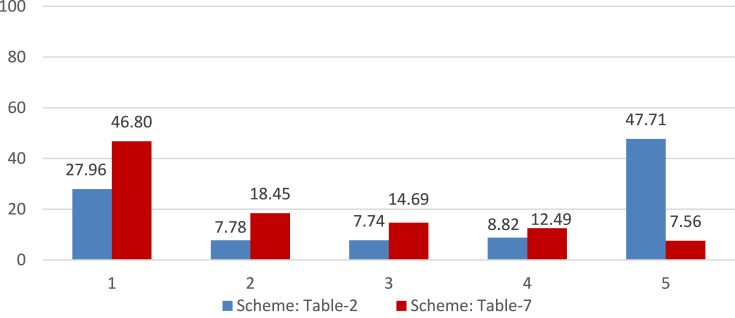
Percentage Distribution of reviewers by their weight in root-set 1(2314 reviewers) using different weight assignment schemes.

**Figure 14:**
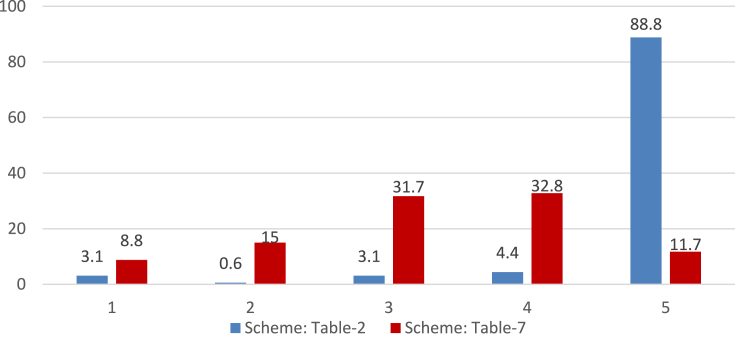
Percentage Distribution of reviewers by their weight in root-set 2(1000 reviewers) using different weight assignment schemes.

To assign and update reviewer's weight based on the reviewer's current average error, alongside the scheme mentioned earlier in Table 2, we experimented with another scheme presented in Table 7 below. As we mentioned earlier, the schemes for determining reviewers' weight are *“subjective and domain-specific”*. The scheme in Table 7 is developed for this smaller dataset to impose a stricter margin of error for assigning weight. This is to accommodate the fact that those majority single-review products cause no error because that one review-rating acts as the true-rating, resulting in less avg. error. The resulted percentage distributions of all the reviewers from the two root sets according to their respective final weight for both the schemes are given in Figures 13 and 14. The scheme in Table 2 assigns higher weights than the scheme in Table 7 as evident from both figures. This is understandable and expected because the scheme in Table 2 was designed for a larger dataset with less single-review products.

**Table 7:** Reviewer's weight according to his Avg. error.

Avg. Error	<0.10	~ <0.20	~ <0.30	~ <0.40	≥0.40
Weight	5	4	3	2	1

The final step of the algorithm involves calculating the score of the reviewers from their weighted avg. error and their weighted number of reviews (using Eq. (8)). Like before, we have performed several trials for various values of *M* to find the 50 experts. Tables 8 and 9 each presents the performance of 30 different sets of potential expert reviewers obtained by varying the value of *M* on two root sets of reviewers. In Table 8, to update reviewers' weights, the scheme in Table 2 was used, whereas in Table 9, the scheme in Table 7 was used to update reviewers’ weight. Tables 10 and 11 present the avg. error and avg. MSE associated with the different sets of expert reviewers presented in Tables 8 and 9, respectively.

**Table 8:** Effect of the influence-constant, *M*, on reviewers' avg. error, avg. MSE, movie coverage and count of reviews (The scheme in Table 2 was used to update users’ weights).

Value of *M*	When experts were derived considering set-1 (Root set: 2314 Reviewers)				When experts were derived considering set-2 (Root set: 1000 Reviewers)			
	No. of reviews made	No. of products covered	Avg. Error	Avg. MSE	No. of reviews made	No. of products covered	Avg. Error	Avg. MSE
0	3817	3791	0.021	0.011	2955	2926	0.028	0.017
25	5800	5679	0.024	0.014	6971	6906	0.033	0.025
50	9185	9029	0.032	0.020	10079	9955	0.041	0.034
75	10907	10668	0.038	0.026	11421	11235	0.043	0.036
100	12159	11916	0.039	0.028	11564	11374	0.043	0.036
200	16216	15817	0.046	0.036	12311	12093	0.046	0.039
300	17007	16606	0.050	0.040	14267	13977	0.048	0.042
400	17234	16811	0.050	0.040	15411	15047	0.052	0.047
500	18498	18041	0.051	0.043	15411	15047	0.052	0.047
1000	19073	18583	0.055	0.046	15950	15549	0.056	0.051
1500	19511	19000	0.058	0.049	16185	15764	0.057	0.052
2000	19776	19269	0.060	0.051	16185	15764	0.057	0.052
3000	20380	19816	0.060	0.053	16185	15764	0.057	0.052
5000	20756	20156	0.062	0.055	16185	15764	0.057	0.052
10000	20798	20177	0.063	0.056	16185	15764	0.057	0.052

**Table 9:** Effect of the influence-constant, *M*, on reviewers' avg. error, avg. MSE, movie coverage and count of reviews (The scheme in Table 7 was used to update users’ weights).

Value of *M*	When experts were derived considering set-1 (Root set: 2314 Reviewers)				When experts were derived considering set-2 (Root set: 1000 Reviewers)			
	No. of reviews made	No. of products covered	Avg. Error	Avg. MSE	No. of reviews made	No. of products covered	Avg. Error	Avg. MSE
0	3932	3910	0.019	0.009	2942	2913	0.027	0.015
25	5815	5694	0.023	0.013	6713	6653	0.034	0.024
50	9289	9136	0.032	0.020	9837	9726	0.042	0.032
75	10539	10296	0.037	0.024	11338	11155	0.046	0.038
100	10980	10730	0.039	0.026	11596	11406	0.047	0.040
200	16343	15942	0.048	0.036	13363	13148	0.050	0.045
300	17032	16620	0.050	0.039	13844	13604	0.051	0.047
400	18697	18252	0.052	0.043	14528	14244	0.053	0.049
500	18920	18454	0.054	0.045	14698	14405	0.054	0.050
1000	19216	18759	0.055	0.047	15440	15077	0.058	0.053
1500	19333	18848	0.057	0.049	15708	15327	0.060	0.056
2000	19333	18848	0.057	0.049	15708	15327	0.060	0.056
3000	19345	18834	0.057	0.048	15708	15327	0.060	0.056
5000	19794	19278	0.060	0.051	15708	15327	0.060	0.056
10000	20438	19840	0.061	0.053	15708	15327	0.060	0.056

**Table 10:** Avg. error and avg. MSE of the sets of expert reviewers from Table 8 based on different true-rating derivation technique.

Value of *M*	Using set-1 (Root set of 2314 Reviewers)						Using set-2 (Root set of 1000 Reviewers)					
	Technique for deriving true product rating						Technique for deriving true product rating					
	Mutual reinforcement		Only review rating		Considering review helpfulness		Mutual reinforcement		Only review rating		Considering review helpfulness	
	Avg. Error	Avg. MSE	Avg. Error	Avg. MSE	Avg. Error	Avg. MSE	Avg. Error	Avg. MSE	Avg. Error	Avg. MSE	Avg. Error	Avg. MSE
0	0.021	0.011	0.038	0.033	0.036	0.030	0.028	0.017	0.064	0.060	0.051	0.045
25	0.024	0.014	0.041	0.033	0.039	0.032	0.033	0.025	0.065	0.061	0.057	0.056
50	0.032	0.020	0.053	0.044	0.052	0.045	0.041	0.034	0.071	0.068	0.066	0.069
75	0.038	0.026	0.059	0.050	0.059	0.053	0.043	0.036	0.071	0.068	0.067	0.071
100	0.039	0.028	0.061	0.053	0.061	0.056	0.043	0.036	0.071	0.069	0.067	0.072
200	0.046	0.036	0.069	0.062	0.069	0.065	0.046	0.039	0.075	0.073	0.072	0.076
300	0.050	0.040	0.072	0.065	0.072	0.070	0.048	0.042	0.076	0.075	0.072	0.077
400	0.050	0.040	0.072	0.065	0.073	0.071	0.052	0.047	0.080	0.080	0.076	0.083
500	0.051	0.043	0.073	0.066	0.073	0.072	0.052	0.047	0.080	0.080	0.076	0.083
1000	0.055	0.046	0.077	0.070	0.076	0.075	0.056	0.051	0.084	0.084	0.082	0.090
1500	0.058	0.049	0.081	0.076	0.081	0.080	0.057	0.052	0.086	0.086	0.083	0.091
2000	0.060	0.051	0.082	0.078	0.082	0.083	0.057	0.052	0.086	0.086	0.083	0.091
3000	0.060	0.053	0.083	0.080	0.083	0.085	0.057	0.052	0.086	0.086	0.083	0.091
5000	0.062	0.055	0.085	0.082	0.085	0.087	0.057	0.052	0.086	0.086	0.083	0.091
10000	0.063	0.056	0.085	0.082	0.085	0.088	0.057	0.052	0.086	0.086	0.083	0.091

**Table 11:** Avg. error and avg. MSE of the sets of expert reviewers from Table 9 based on different true-rating derivation technique.

Value of *M*	Using set-1 (Root set of 2314 Reviewers)						Using set-2 (Root set of 1000 Reviewers)					
	Technique for deriving true product rating						Technique for deriving true product rating					
	Mutual reinforcement		Only review rating		Considering review helpfulness		Mutual reinforcement		Only review rating		Considering review helpfulness	
	Avg. Error	Avg. MSE	Avg. Error	Avg. MSE	Avg. Error	Avg. MSE	Avg. Error	Avg. MSE	Avg. Error	Avg. MSE	Avg. Error	Avg. MSE
0	0.019	0.009	0.038	0.032	0.035	0.029	0.027	0.015	0.062	0.058	0.049	0.043
25	0.023	0.013	0.042	0.035	0.040	0.033	0.034	0.024	0.067	0.064	0.059	0.058
50	0.032	0.020	0.054	0.045	0.052	0.046	0.042	0.032	0.070	0.065	0.065	0.066
75	0.037	0.024	0.058	0.049	0.058	0.051	0.046	0.038	0.073	0.069	0.069	0.072
100	0.039	0.026	0.060	0.051	0.060	0.053	0.047	0.040	0.074	0.072	0.070	0.075
200	0.048	0.036	0.070	0.063	0.070	0.066	0.050	0.045	0.077	0.078	0.073	0.080
300	0.050	0.039	0.071	0.064	0.072	0.070	0.051	0.047	0.076	0.078	0.072	0.080
400	0.052	0.043	0.073	0.067	0.073	0.073	0.053	0.049	0.077	0.078	0.074	0.080
500	0.054	0.045	0.074	0.068	0.074	0.074	0.054	0.050	0.078	0.078	0.074	0.080
1000	0.055	0.047	0.075	0.070	0.076	0.077	0.058	0.053	0.083	0.082	0.079	0.086
1500	0.057	0.049	0.078	0.072	0.078	0.080	0.060	0.056	0.084	0.084	0.081	0.088
2000	0.057	0.049	0.078	0.072	0.078	0.080	0.060	0.056	0.084	0.084	0.081	0.088
3000	0.057	0.048	0.078	0.072	0.078	0.078	0.060	0.056	0.084	0.084	0.081	0.088
5000	0.060	0.051	0.080	0.076	0.080	0.082	0.060	0.056	0.084	0.084	0.081	0.088
10000	0.061	0.053	0.082	0.078	0.082	0.084	0.060	0.056	0.084	0.084	0.081	0.088

We observe in Table 6 that the lowest avg. error and avg. MSE was obtained by the SFS algorithm. In terms of product coverage and the number of reviews made, the expert reviewers found through the SFS algorithm covered nearly 9 thousand products and made a similar number of reviews (8765 by SFS_40 and 9144 by SFS_50). Table 8 shows that similar coverage is found when *M* is set to 75 for set-1 and 50 for set-2 of potential experts while using the scheme in Table 2 to update reviewers' weight. The recommended 50 experts by this mutual reinforcement technique in Table 8 cover 10668 and 9955 products and made 10907 and 10079 reviews respectively. Table 9 is similar to Table 8 while using the scheme in Table 7 to update the reviewers’ weight. In Table 9, when *M* is set to 75 for set-1 and 50 for set-2 of potential experts, the recommended 50 experts cover 10296 and 9726 products and made 10539 and 9837 reviews respectively.

Table 12 shows that all four sets of reviewers recommended by the mutual reinforcement technique have lower average error and lower average MSE than the set of reviewers recommended by SFS. The best among these four sets is from set-1 with M = 75, and the worst is from set-2 with M = 50. Compared to the SFS algorithm, the best set of recommended experts reduced the avg. error and avg. MSE by (i) 43.94% and 47.83%, (ii) 39.58% and 37.18%, and (iii) 36.26% and 32.89% respectively when product's true rating is estimated by (i) mutual reinforcement, (ii) an average of review ratings, and (iii) weighted average of review ratings considering helpfulness votes respectively. The worst among these four sets of recommended experts reduced the avg. error and avg. MSE by 36.36% and 26.09%, 26.04% and 12.82%, and 27.47% and 9.21% respectively. All four sets of reviewers covered more products and made more reviews than the reviewers recommended by SFS.

**Table 12:** Performance comparison between experts of SFS algorithm and different candidates from *mutual reinforcement* algorithm.

Method of expert reviewer detection			True product rating using mutual reinforcement		True product rating using only review ratings		True product rating Considering review helpfulness	
			Avg. Error	Avg. MSE	Avg. Error	Avg. MSE	Avg. Error	Avg. MSE
SFS_40			0.066∗	0.046	0.096	0.078	0.091	0.076
SFS_50			0.067∗	0.048	0.097	0.080	0.092	0.077
Mutual reinforcement	Set-1 *M* = 75	Table 8	0.038	0.026	0.059	0.050	0.059	0.053
		Table 9	0.037	0.024	0.058	0.049	0.058	0.051
	Set-2 *M* = 50	Table 8	0.041	0.034	0.071	0.068	0.066	0.069
		Table 9	0.042	0.032	0.070	0.065	0.065	0.066

∗ Taking the smallest avg. error among the four ‘True product rating using mutual-reinforcement’ variations used in Tables 8 and 9.

**Figure 15:**
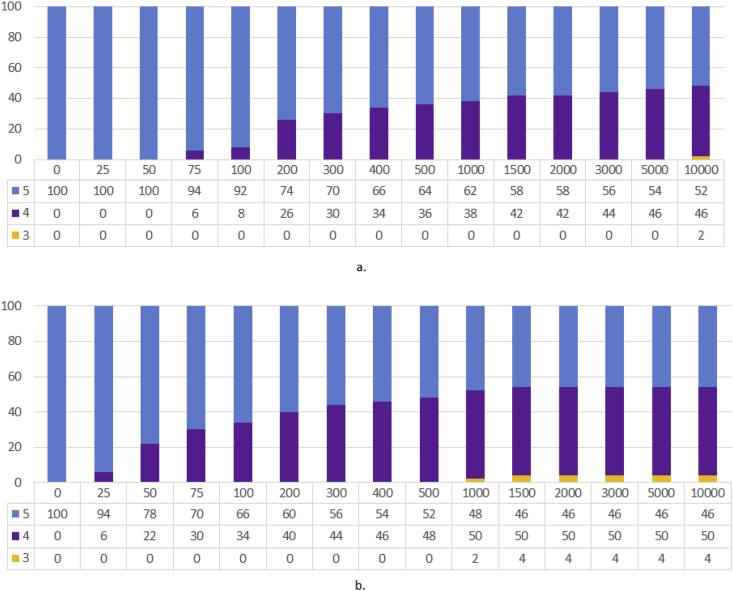
Percentage distribution of the various sets of 50 recommended experts (from Table 9) by their weight. a. Different sets of recommende experts for different values of M from set-1. b. Different sets of recommende experts for different values of M from set-2.

From Tables 8, 9, 10, and 11 and Figure 15, we observe *M*'s impact as expected; lower values of *M* produce sets of experts with lower coverage of movies and review-count, and lower avg. error. The absence/low participation of reviewers of weight 1–3 is noticeable. We have not presented the weight-distribution figure for Table 8 because all 50 of the recommended reviewers were of weight 5 irrespective of the value of M. The reason for this absence/low participation of other weight-category reviewers is that the avg. error in this smaller dataset is so low in terms of the weight-division scheme for updating reviewers' weight that a significant portion of them (in some cases, all) ends up in higher weight categories (demonstrated in Figures 13 and 14).

### 4.4 Experimentation and result: dataset 3

In this dataset, there were 23965 reviewers. Among them, 1455 reviewers made more than 50 reviews, 1738 reviewers made more than 40 reviews, and 2195 reviewers made more than 30 reviews. We limit our search for experts among the 2195 reviewers who made more than 30 reviews each. Like the previous experiment, our goal is to find 50 experts from them which is roughly 2.3% of the eligible reviewers.

#### 4.4.1 Performance of different straightforward techniques and SFS algorithm

**Figure 16:**
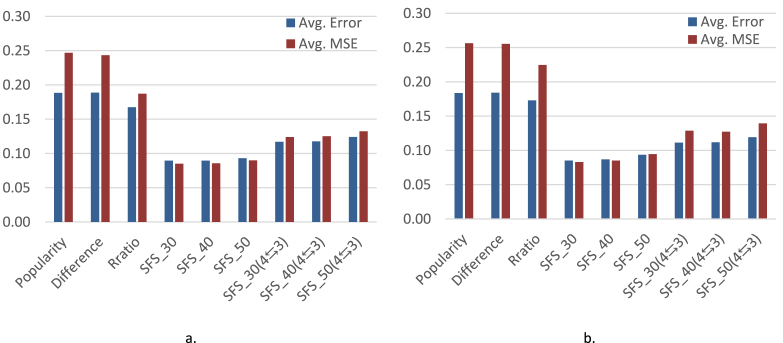
Avg. error and avg. MSE of the different expert reviewer detection techniques. a. True Rating considering only review-rating. b. True Rating considering helpulness votes.

The customized SFS algorithm (Figure 10) applied on dataset 2 was applied to this dataset as well. The only exception here is that an additional initial set of potential experts was created using a lower eligibility threshold of 30 reviews. Table 13 and the column-charts in Figure 16 below show the performance of the different expert reviewer detection techniques.

**Table 13:** Performance of the different expert reviewer detection techniques.

Method of expert reviewer detection	Product's True Rating considering only review-rating (Eq. (1))				Product's True Rating considering helpfulness votes (Eq. (5))			
	No. of reviews made	No. of products covered	Avg. Error	Avg. MSE	No. of reviews made	No. of products covered	Avg. Error	Avg. MSE
Popularity	129601	116621	0.188	0.247	129601	116621	0.184	0.256
Difference	59213	54782	0.189	0.243	59213	54782	0.184	0.255
Help-ratio	2848	2807	0.167	0.187	2848	2807	0.173	0.225
SFS_30	15047	14932	0.089	0.085	13950	13870	0.085	0.083
SFS_40	15729	15619	0.090	0.086	14098	14021	0.087	0.085
SFS_50	16827	16661	0.093	0.090	15958	15844	0.094	0.095
SFS_30 (step 4⇆3)	9244	9136	0.117	0.124	8010	7927	0.111	0.129
SFS_40 (step 4⇆3)	7921	7833	0.118	0.125	8160	8090	0.112	0.127
SFS_50 (step 4⇆3)	9449	9337	0.124	0.132	9202	9102	0.119	0.140

Table 13 and the column-charts in Figure 16 show that the SFS technique outperforms the usual straightforward methods again. When the product rating is calculated considering only the review-rating, its recommended experts achieved almost 46% {(0.167−0.089)/(0.167)} reduction in average error and 54% {(0.187−0.085)/(0.187)}reduction in MSE than the best straightforward technique. When the product rating is calculated considering the helpfulness votes, the experts achieved almost **50%** {(0.173−0.085)/(0.173)} reduction in average error and **63%** {(0.225−0.083)/(0.225)} reduction in MSE.

#### 4.4.2 Expert reviewer detection by mutual reinforcement

Similar to the previous experiment, we constructed two root sets of potential experts in step-1. Set-1 was created by selecting those reviewers who have made more than 30 reviews each. Set-2 was created by selecting the top 1000 reviewers who have the largest thumb-ups to the thumb-downs difference from set-1. There are 369652 products associated with the set-1 reviewers and 216943 products associated with set-2 reviewers. However, the smallness of the dataset is revealed by the fact that more than 70% of the associated products in both sets have only a single review each. In step-2 of this algorithm, we assign weights to products following the same procedure used in the previous experiment (see 4.3.2). The weight distribution of the products is shown in Table 14.

**Table 14:** Distribution of instant video products according to weight.

Weight	1	2	3	4	5	6	7	8	9	10
Entire Dataset	421850	3985	644	206	87	54	27	20	8	41
Products in set-1	364728	3867	627	202	84	53	24	19	8	40
Products in set-2	212278	3645	607	191	82	52	22	19	8	39

To update the reviewer's weight based on the reviewer's current average error, we used the same scheme shown in Table 7, which was applied to the previous experiment as well. The final step of the algorithm involves calculating the score of the reviewers from their weighted avg. error and their weighted number of reviews (using Eq. (8)). Like before, we have performed several trials for various values of *M* to find the 50 experts. Table 15 and Table 16 present the performance of 30 different sets of potential experts obtained by varying the *M*'s value on the two root sets of potential expert reviewers.

**Table 15:** Effect of the influence-constant, *M*, on reviewers’ avg. error, avg. MSE, movie coverage and count of reviews.

Value of *M*	When experts were derived considering set-1 (Root set: 2195 Reviewers)				When experts were derived considering set-2 (Root set: 1000 Reviewers)			
	No. of reviews made	No. of products covered	Avg. Error	Avg. MSE	No. of reviews made	No. of products covered	Avg. Error	Avg. MSE
0	3539	3510	0.022	0.014	4329	4226	0.032	0.021
25	11875	11644	0.034	0.027	15695	15374	0.037	0.029
50	22519	21681	0.046	0.040	26792	25990	0.048	0.042
75	35186	33709	0.056	0.053	29236	28339	0.054	0.049
100	36292	34669	0.058	0.055	33414	32410	0.059	0.053
200	55437	52858	0.069	0.069	38995	37680	0.063	0.060
300	68055	64793	0.076	0.078	41284	39849	0.068	0.065
400	71506	67931	0.078	0.080	43522	41988	0.071	0.068
500	74250	70531	0.080	0.085	44507	42862	0.072	0.071
1000	81006	76289	0.086	0.092	55264	53016	0.078	0.078
1500	101545	94273	0.091	0.100	55551	53264	0.079	0.078
2000	106693	98115	0.094	0.104	55551	53264	0.079	0.078
3000	117574	108534	0.097	0.108	55985	53643	0.079	0.079
5000	118327	109305	0.098	0.109	55985	53643	0.079	0.079
10000	122062	112510	0.101	0.114	64448	61830	0.082	0.083

**Table 16:** Avg. error and avg. MSE of the sets of expert reviewers from Table 15 based on different true-rating derivation technique.

Value of *M*	Using set-1 (Root set of 2195 Reviewers)						Using set-2 (Root set of 1000 Reviewers)						
	Technique for deriving true product rating						Technique for deriving true product rating						
	Mutual reinforcement		Only review rating		Considering review helpfulness		Mutual reinforcement		Only review rating		Considering review helpfulness		
	Avg. Error	Avg. MSE	Avg. Error	Avg. MSE	Avg. Error	Avg. MSE	Avg. Error	Avg. MSE	Avg. Error	Avg. MSE	Avg. Error	Avg. MSE	
0	0.022	0.014	0.044	0.045	0.037	0.034	0.032	0.021	0.068	0.077	0.059	0.065	
25	0.034	0.027	0.056	0.056	0.053	0.053	0.037	0.029	0.067	0.077	0.063	0.075	
50	0.046	0.040	0.066	0.066	0.065	0.070	0.048	0.042	0.078	0.088	0.075	0.086	
75	0.056	0.053	0.074	0.077	0.075	0.086	0.054	0.049	0.082	0.089	0.082	0.097	
100	0.058	0.055	0.075	0.079	0.076	0.090	0.059	0.053	0.086	0.093	0.087	0.102	
200	0.069	0.069	0.086	0.092	0.089	0.108	0.063	0.060	0.091	0.099	0.092	0.110	
300	0.076	0.078	0.093	0.100	0.097	0.119	0.068	0.065	0.095	0.104	0.097	0.119	
400	0.078	0.080	0.094	0.102	0.099	0.121	0.071	0.068	0.100	0.110	0.102	0.126	
500	0.080	0.085	0.096	0.104	0.101	0.124	0.072	0.071	0.100	0.110	0.103	0.128	
1000	0.086	0.092	0.101	0.109	0.107	0.131	0.078	0.078	0.106	0.117	0.109	0.136	
1500	0.091	0.100	0.106	0.116	0.112	0.138	0.079	0.078	0.106	0.116	0.110	0.136	
2000	0.094	0.104	0.108	0.117	0.115	0.142	0.079	0.078	0.106	0.116	0.110	0.136	
3000	0.097	0.108	0.111	0.120	0.118	0.148	0.079	0.079	0.106	0.116	0.111	0.136	
5000	0.098	0.109	0.112	0.122	0.119	0.149	0.079	0.079	0.106	0.116	0.111	0.136	
10000	0.101	0.114	0.115	0.125	0.122	0.153	0.082	0.083	0.108	0.118	0.113	0.140	

**Figure 17:**
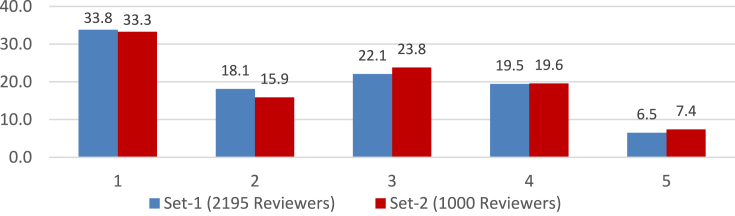
Percentage Distribution of reviewers in the two root sets according to their weight.

The percentage distribution of all the reviewers from the two root sets according to their respective final weight is given in Figure 17. Understandably the distribution is heavier towards the lower weights.

We observe in Table 13 that the lowest avg. error and avg. MSE was obtained by the SFS algorithm. When true product ratings are derived considering only review-ratings, their lowest avg. error and avg. MSE are 0.089 and 0.085 respectively. When true product ratings are derived considering the helpfulness votes of the review, the lowest avg. error and avg. MSE are 0.085 and 0.083 respectively. Regarding product coverage and the number of reviews, the expert reviewers found through the best SFS variation covered 14932 products and made 15047 reviews when true product ratings are derived considering only review-ratings (13870 products and 13950 reviews, when true product ratings are derived considering helpfulness votes). Table 15 shows that when *M* is set to 50 for set-1 and 25 for set-2 of potential experts, the recommended 50 experts by this *mutual reinforcement technique* cover 21681 and 15695 products and made 22519 and 15695 reviews respectively. The following Table 17 compares their avg. error and avg. MSE.

**Table 17:** Performance comparison between experts of SFS algorithm and different candidates from *mutual reinforcement* algorithm.

Method of expert reviewer detection		True product rating using mutual reinforcement		True product rating using only review ratings		True product rating considering review helpfulness	
		Avg. Error	Avg. MSE	Avg. Error	Avg. MSE	Avg. Error	Avg. MSE
SFS_30		0.064∗	0.054∗	0.089	0.085	0.085	0.083
Mutual reinforcement	Set-1, *M* = 50	0.046	0.040	0.066	0.066	0.065	0.070
	Set-2, *M* = 25	0.037	0.029	0.067	0.077	0.063	0.075

∗ Taking the smallest value among the two ‘True product rating using mutual-reinforcement’ variations used in Table 15.

Table 17 shows that both sets of reviewers recommended by the mutual reinforcement technique cause less average error and less average MSE than the recommended reviewers by SFS. Compared to the SFS algorithm, the recommended experts from set-1 with M = 50 reduced the avg. error and avg. MSE by (i) 28.13% and 25.93%, (ii) 25.84% and 22.35%, and (iii) 22.53% and 15.66% respectively when product's true rating is estimated respectively by (i) mutual reinforcement, (ii) an average of review ratings, and (iii) weighted average of review ratings considering helpfulness votes. The recommended experts from set-2 with M = 25 reduced the avg. error and avg. MSE by 42.19% and 46.30%, 24.72% and 9.41%, and 25.88% and 9.64% respectively.

**Figure 18:**
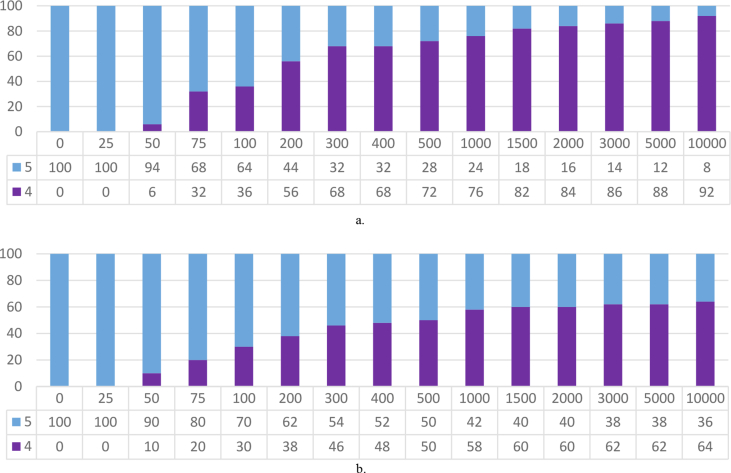
Percentage distribution of the various sets of 50 recommended experts (from Table 15) by their weight. a. Different sets of recommended experts for different values of M from set-1. b. Different sets of recommended experts for different values of M from set-2.

From Figure 18 below, we observe that the recommended experts are limited to weight-class 4 and 5. Figure 17 showed that these two classes consists of more than 25% of the potential experts. This large share of higher weight-classes is the primary reason for absence of reviewers of lower weight classes (in contrast, see Figures 7, 8, and 9 where higher weight-classes are smaller compared to the eligible population).

### 4.5 Discussion regarding the experiments

Three different domains of experiential products were chosen to ensure the inclusion of inherent variability (e.g. size of the dataset, level of engagement of the reviewers, number of reviews per product etc.) of the domains. As one of the largest e-commerce sites globally, following the law of large number, the first large dataset of reviews is expected to capture the characteristics of a standard review-community. In terms of reviews, the second dataset is almost one-tenth the size of the first one. The third dataset is even smaller, consisting of 23965 reviewers and 583933 reviews. All three datasets spanned for more than ten years. In all three experiments, the second algorithm based on mutual reinforcement was found superior to the other algorithms both for detecting experts and deriving product ratings (reducing the avg. error and avg. MSE while maintaining similar product coverage and number of reviews).

The two proposed algorithms have several parameters that need to be adjusted for the underlying domain. For the reinforcement-based algorithm, the adjustable parameters that impact the set of reviewers are: (i) the criteria to get included in the initial set of potential experts, (ii) the scheme for assigning weights to products based on the no. of reviews, (iii) the scheme for assigning weights to reviewers based on respective avg. error, and (iv) value of the influence constant-*M*. Proper adjustment of these parameters will lead to the reduction of avg. error and avg. MSE. The concept of mutual reinforcement inherently ensures that a product's rating leans towards heavy-weighted reviewers, and a product's weight impacts the reviewer's weight. This mechanism itself lowers the avg. error and avg. MSE. However, to make sure that mutual reinforcement takes place, the adjustable parameters need to be tuned appropriately considering the nature of the underlying environment. For example, a relatively low/high threshold for assigning weights to products or reviewers will cause most products or reviewers to end up in the same high/low weight class, compromising the mutual reinforcement mechanism. Similarly, the value of influence-constant *M* should be adjusted appropriately. For example, the best result for dataset-1 was obtained when *M* was set to 700. However, for the second and third dataset, the best results were achieved when *M* was set in the range of 25–75. In the SFS algorithm, the parameters that impact the outcome are: (i) the criteria to get included in the initial set of potential experts, (ii) the size of the second, third, and fourth stages with respect to the required quantity of expert reviewers.

In the experiments, the inclusion criteria in the initial set of potential experts were varied in line with the community's engagement. The aim was to create a root set with a large enough quantity of engaged reviewers. If we think that 5% within an engaged population acquires expert-level skill in a certain trade, then to get 5 experts, the initial set has to contain at least 100 engaged candidates (20 times larger). In the experiments, the applied thresholds were 50, 40 and 30 reviews, which ensure that the initial sets of potential experts are at least 30 times larger than the targeted number of experts. In the SFS algorithm, the size of the second, third and fourth stages were also varied in line with the targeted number of experts. Alongside these formations, we also tested with another smaller potential set (referred to as set-2 in the experiments) based on the helpfulness difference. This set-2 was at least 10 times larger than the targeted number of experts.

Regarding the schemes to update reviewers' and products’ weight, the outcome of different schemes was graphically presented and inspected (in figures depicting the distribution of products/reviewers by weight) to ensure mutual reinforcement was taking place. Finally, 15 different values of *M* were used to observe the difference in performance and to select the best value for *M*. A crude analogy of these adjustable parameters to the proposed algorithms would be k-means algorithm where the value of *k* and the initial *k*-seeds influence the result. All three datasets used in the experimentations are publicly available. The study is completely reproducible.

### 4.6 Regarding the treatment of star-rating and likert-type data

It should be noted that there exists controversy regarding the usage of metric/parametric statistics on Likert/Likert-type data. For example, Kuzon et al. [32] wrote that the 1st deadly sin of statistical analysis is using parametric statistics on ordinal data. Highlighting the seemingly unequal intervals between anchors Jamieson [33] wrote ‘‘the appropriate descriptive and inferential statistics differ for ordinal and interval variables and if the wrong statistical technique is used, the researcher increases the chance of coming to the wrong conclusion’’. On the other hand, many researchers are proponent of the opposite view (e.g. Norman [34] and Carifio [35]) and demonstrated the effectiveness of metric/parametric statistics on Likert-type data. Professor Harpe [36] mentioned several recent studies that found the presence of a “mental number line” in humans when presented with numbers, implicating that “the intervals between numbers may actually be equal since they appear to be mapped to this internal number line.” Star-ratings have become so ubiquitous nowadays that some websites present it to the users without mentioning any anchor (e.g., flipcart.com) and some websites present the anchors secondarily after a user selects the number of stars (e.g., amazon.com, Google's rating of businesses/places on map).

A large part of the community of researchers have been using metric/parametric statistics on Likert-type data. For example, in the 2016 volumes of the Journal of Personality and Social Psychology (JPSP), Psychological Science (PS), and the Journal of Experimental Psychology: General (JEP:G), all of the 68 articles that use the term ‘Likert’ treated the ordinal data as metric and used a metric model [37]. Edmondson et al. [38] explored the Journal of Marketing (JM) from its inception till 2007 and found 53.70% of the 311 studies (from 251 articles selected for the study) used the mean as the “central tendency measure” and 44.69% of these studies used standard deviation as the “variability measure”.

## 5 Conclusion and future work

Online retailers have already established themselves as a strong competitor of the traditional brick and mortar stores. These retailers support their potential customers with product information by building online communities to provide product reviews to customers. The credibility of these reviews is a decisive factor when purchasing a product. In real life too, we tend to attach different weights to different sources according to their social-ties and expertise level. Finding and highlighting the experts will be a great help to consumers in this regard.

In this study, we first presented a mixed adaptation of the open-source techniques, which performed better than the individual techniques. Then we proposed a second algorithm which is based on the HITS link analysis algorithm. We performed a large scale evaluation of these methods demonstrating their effectiveness for detecting experts. We found that the expert reviewers suggested by the proposed second algorithm made significant improvement in terms of avg. error and avg. MSE while maintaining similar product coverage and number of reviews.

Our contributions are as follows:

• We proposed two different new models and several variations for detecting the expert reviewers.
• We proposed two different techniques for deriving the product ratings.
• We compared the performance of different algorithms on three datasets employing all three product rating techniques and found proposed link-analysis based model significantly superior over the other models, both for detecting experts and deriving product ratings.
• This is the first open work, to our knowledge, to detect expert reviewers and derive product ratings applying link analytic techniques over this type of reviewer community.

There are still areas to explore in the future. When deriving the product rating and detecting the experts, we did not use the review-text in any of our proposed algorithms. We relied on the helpfulness votes and mutual reinforcement between reviewers and products for those purposes. However, analyzing the review-text to predict the product rating and review helpfulness is an active area of research [39, 40, 41, 42, 43, 44, 45]. In the future, the predicted helpfulness of reviews suggested by these text-mining techniques may be compared and incorporated in the algorithm to determine the combined (including the thumb-up/down votes) helpfulness of the reviews, and consequently, the ratings of the products and the rank of the reviewers.

## Declarations

### Author contribution statement

A. R. Sarkar: Conceived and designed the experiments; Performed the experiments; Analyzed and interpreted the data; Contributed reagents, materials, analysis tools or data; Wrote the paper.

S. Ahmad: Conceived and designed the experiments; Analyzed and interpreted the data; Wrote the paper.

### Funding statement

This work was partially supported by a Fellowship from ICT Division, Government of the People's Republic of Bangladesh (Grant No. 56.00.00000.028.33.007.14-257).

### Data availability statement

Data associated with this study has been deposited at https://data.mendeley.com/datasets/kb5nv7dbtm/1 and http://jmcauley.ucsd.edu/data/amazon/links.html.

### Declaration of interests statement

The authors declare no conflict of interest.

### Additional information

No additional information is available for this paper.
